# Incidence of Apical Crack Initiation during Canal Preparation using Hand Stainless Steel (K-File) and Hand NiTi (Protaper) Files

**DOI:** 10.5005/jp-journals-10005-1382

**Published:** 2016-12-05

**Authors:** Dileep Soni, Deepak Raisingani, Rachit Mathur, Nidha Madan, Suchita Visnoi

**Affiliations:** 1 Student, Department of Conservative Dentistry and Endodontic Mahatma Gandhi Dental College and Hospital, Jaipur, Rajasthan India; 2 Postgraduate Student, Department of Conservative Dentistry and Endodontic Mahatma Gandhi Dental College and Hospital, Jaipur, Rajasthan India; 3 Senior Lecturer, Department of Conservative Dentistry and Endodontic Mahatma Gandhi Dental College and Hospital, Jaipur, Rajasthan India; 4 Professor and Head, Department of Conservative Dentistry and Endodontic Mahatma Gandhi Dental College and Hospital, Jaipur, Rajasthan India; 5 Senior Lecturer, Department of Conservative Dentistry and Endodontic Mahatma Gandhi Dental College and Hospital, Jaipur, Rajasthan India

**Keywords:** Cracks, Instrumentation, Root canal preparation, Vertical root fracture, Working length.

## Abstract

**Aim:**

To evaluate the incidence of apical crack initiation during canal preparation with stainless steel K-files and hand protaper files *(in vitro* study).

**Materials and methods:**

Sixty extracted mandibular premo-lar teeth are randomly selected and embedded in an acrylic tube filled with autopolymerizing resin. A baseline image of the apical surface of each specimen was recorded under a digital microscope (80×). The cervical and middle thirds of all samples were flared with #2 and #1 Gates-Glidden (GG) drills, and a second image was recorded. The teeth were randomly divided into four groups of 15 teeth each according to the file type (hand K-file and hand-protaper) and working length (WL) (instrumented at WL and 1 mm less than WL). Final image after dye penetration and photomicrograph of the apical root surface were digitally recorded.

**Results:**

Maximum numbers of cracks were observed with hand protaper files compared with hand K-file at the WL and 1 mm short of WL. Chi-square testing revealed a highly significant effect of WL on crack formation at WL and 1 mm short of WL (p = 0.000).

**Conclusion:**

Minimum numbers of cracks at WL and 1 mm short of WL were observed with hand K-file and maximum with hand protaper files.

**How to cite this article:**

Soni D, Raisingani D, Mathur R, Madan N, Visnoi S. Incidence of Apical Crack Initiation during Canal Preparation using Hand Stainless Steel (K-File) and Hand NiTi (Protaper) Files. Int J Clin Pediatr Dent 2016;9(4):303-307.

## INTRODUCTION

The goals of endodontic instrumentation are to completely remove debris and tissues by enlarging the canal diameter and create a canal form that allows a proper seal.^1-3^ However, preparation procedures could also damage the root dentin, resulting in fractures or craze lines.^3^

Various studies suggest that vertical root fracture (VRF) is probably not an instant phenomenon, but rather a result of gradual diminution of root structure.^3^ The results could confirm that fractures did not occur immediately after canal preparation. However, craze lines occurred in 4 to 16%, and these may develop into fractures during retreatment or after long-term functional stresses like chewing. This is in agreement with Wesselink et al,^3^ who were the first to report dentinal defects as a consequence of canal preparation, but only found small defects entirely within dentin that did not communicate with the canal wall. Considering the crucial clinical importance of VRF and its determinant effect on tooth survival, even a small percentage of damaged teeth could be of clinical impor-tance.^3^ A prepared canal that eliminates these narrow extensions will have a more uniform stress distribution and potentially a much reduced susceptibility to fracture. It is generally accepted that the strength of an endodonti-cally treated tooth is directly related to the amount of the remaining sound tooth structure.

Several treatment procedures, such as caries removal, access preparation, instrumentation of the root canal, irrigation of the canal with sodium hypochlorite, and long-term intracanal dressings with calcium hydroxide lead to a loss of tooth structure or may weaken the dentin. Traditionally, root canal preparation was carried out using stainless steel endodontic files manipulated by hand. Advances in rotary nickel-titanium instruments have led to new designs and techniques of root canal preparation.^4^ NiTi instruments are believed to allow preparations of root canals with fewer procedural errors than conventional stainless steel instruments.^5^ Complete planning of canal walls and hence a completely smooth canal shape and taper remains an elusive ideal.^6^ In a study by Bier et al, flexible K-files with a small taper caused significantly less cracks than large taper. Instrumentation to apical foramen could cause more apical root cracks than instrumentation short of apical foramen. The purpose of the present study was to compare the incidence of root cracks observed at the apical root surface and/or in the canal wall after root canal instrumentation with protaper and stainless steel K-files at working length (WL) and 1 mm short of WL.^5,6^

## MATERIALS AND METHODS

### Collection of Samples and Storage

**Fig. 1:**
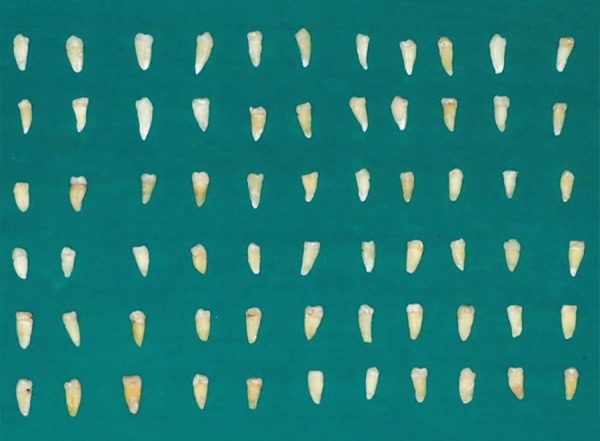
Extracted mandibular permanent premolar teeth

**Fig. 2:**
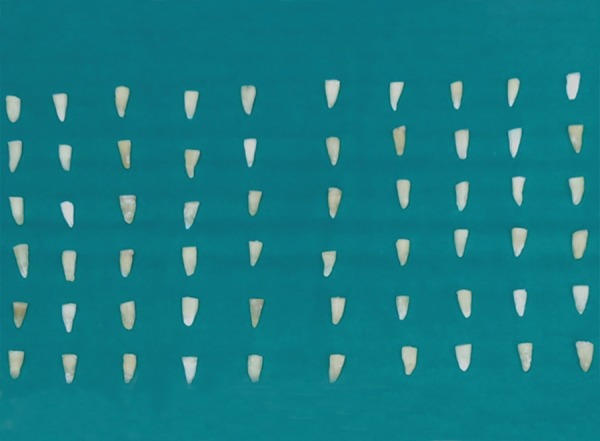
Decoronated sections of roots

**Fig. 3:**
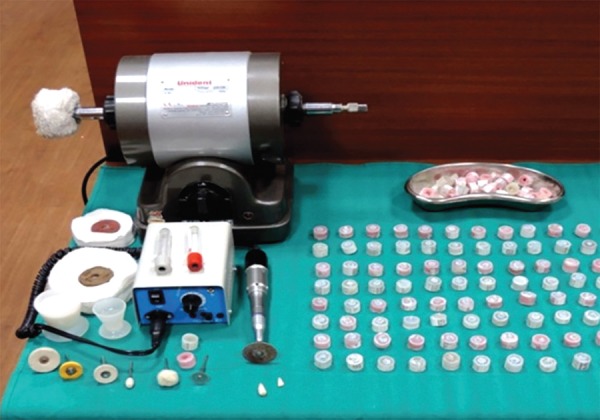
Prepared cylindrical acrylic molds

**Fig. 4:**
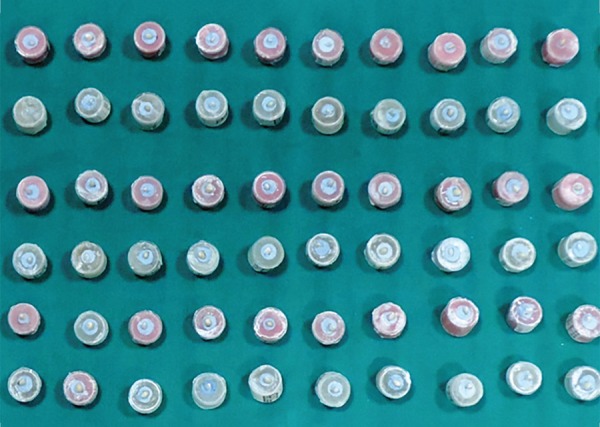
Apical surface in cylindrical molds

**Fig. 5:**
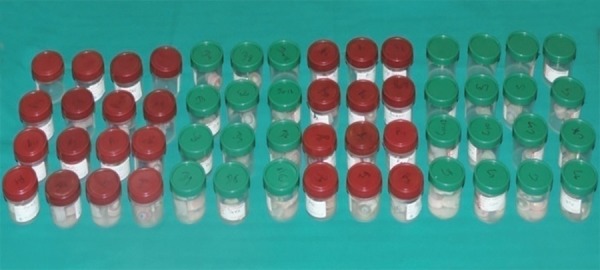
Storage of sample

A total of 60 freshly extracted human single-rooted permanent mandibular premolars were collected (Fig. 1). To standardize the root length samples, teeth were deco-ronated using diamond disk in a low-speed micromotor straight handpiece. The crown of each tooth was resected 2 mm coronal to the cementoenamel junction to facilitate straight line access for instrumentation (Fig. 2). The flat surface 2 mm above the cementoenamel junction was used as the reference plane. The distance between the reference plane and the tip of the file was defined as the WL (= root canal length). The teeth were wrapped with a single layer of aluminum foil and were embedded in autopolymerizing resin set in an acrylic tube (12 mm high and 16 mm in diameter). The teeth were then removed from the tube, and the aluminum foil was peeled off. The root surface and the socket were then coated with a hydrophilic vinyl polysiloxane impression material, and the teeth were immediately repositioned. Thus, the polysiloxane replaced the space created by the foil (Figs 3 and 4). The samples were then stored in distilled water at room temperature until use (Fig. 5).

### Method of Instrumentation

Working length was determined by a size 10 stainless steel K-file until it became visible at the apex. Root canals were prepared using crown down technique. The coronal 1/3rd of the canal was prepared with Gates-Glidden (GG) drills (No. 1 and 2). All canals were irrigated with 3% sodium hypochlorite solution during and after instrumentation. A total of 0.9% normal saline with ethylene-diaminetetraacetic acid was used for cleaning the root canal. Apical patency was maintained with a stainless steel size 15 K-file, followed by recapitulation with a size 15 file after each file change. Teeth were stored in distilled water after the instrumentation procedure to prevent dehydration. The teeth were randomly distributed into four experimental groups of 15 teeth per group.

**Figs 6A and B:**
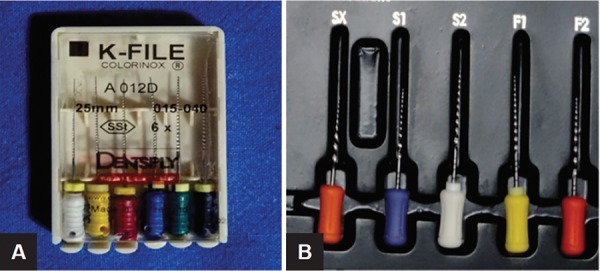
Files used in the study: (A) Hand K-file; (B) hand protaper file

### Group I (at WL)

Teeth were instrumented with hand stainless steel K-file. File size 15 K was used to prepare the canal until the complete WL. The canals were instrumented sizes 20, 25 until the root canal length, and analysis of the crack was made (Figs 6A and B).

### Group II (1 mm less WL)

Stainless steel K-files, same as group I, except that the samples were prepared 1 mm less of WL.

### Group III (at WL)

**Fig. 7:**
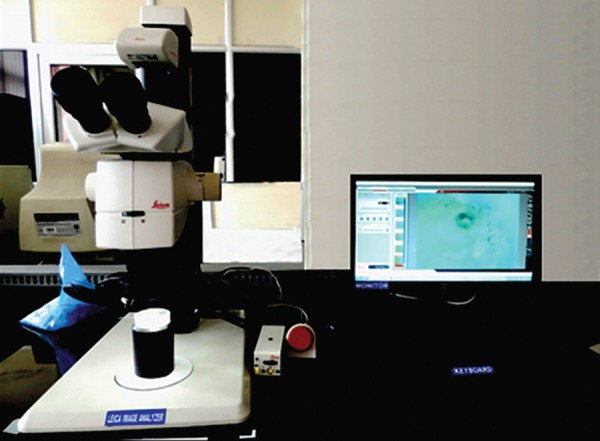
Digital microscope (80×)

Teeth were instrumented with hand protaper files. The canals were instrumented following the sequence of sizes S1, S2, F1, F2 until the root canal length, and analysis of the crack was made (Fig. 7).

### Group IV (1 mm less WL)

Hand protaper files, same as group I, except that the samples were prepared 1 mm less of WL.

## EVALUATION

“A crack is defined as any visible discontinuity that originated from the root canal. The images were examined for the presence and absence of cracks on apical root surface.”

**Fig. 8:**
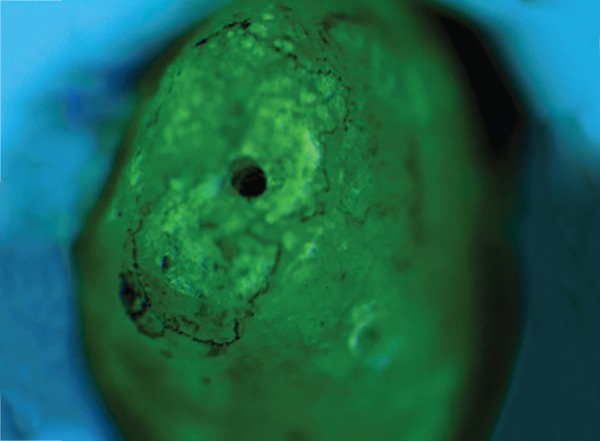
Apical crack in tooth (in digital microscope 80×)

Each sample in the acrylic block was placed under a digital microscope [(Leica Microsystems, Wetzlar, Germany 80×) (Fig. 7)] to record the standardized photomicrograph images of the root apex. This image was used as the baseline image (first image). Second image was after instrumentation of coronal 1/3rd by GG drills, and photomicrograph of the apical root surface was digitally recorded. The samples were divided into four groups of 15 teeth each for canal preparation at different instrumentation lengths. After final instrumentation, photomicrograph (third image) of the apical root surface was recorded (at WL and 1 mm short of WL). Photomicrograph (final image) was digitally recorded after application of India Ink dye (Fig. 8). For the final analysis, the images were stored in Office PowerPoint 2007 (Microsoft Corporation, Seattle, WA, USA), and each slide was coded. It was used to create a slideshow presentation with the four images per sample per slide.

### Statistical Analysis

Data were analyzed using chi-square test to determine variances among all groups. All statistical analysis was performed at 95% level of confidence.

**Table 1:** **Table 1:** Total number of teeth with cracks on hand K-file and hand protaper file

*Group*		*At WL (n = 15)*		*1 mm short of* *WL (n = 15)*	
Hand K-file		8		1	
Hand protaper file		12		2	

**Table 2:** **Table 2:** Chi-square testing

		*Cracks*							
*WL*		*Present*		*Absent*		*χ^2^* *(df)*		*p-value*	
At WL (n = 30)		20		10		20.4 (1)		0.000	
At 1 mm short of		3		27					
WL (n = 30)									

**Graph 1:**
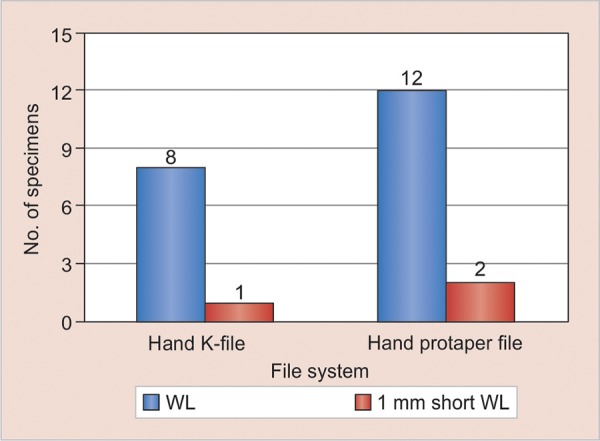
Total number of teeth with cracks on hand K-file and hand protaper file

## RESULTS

Table 1 shows that more apical root cracks were found with hand protaper file (at WL and or 1 mm short of WL) and least with hand K-file. Table 2 shows Chi-square testing that revealed a highly significant effect of WL on crack formation at WL and 1 mm short of WL (p = 0.000). The results indicate that a crack is more likely to appear at WL compared with 1 mm short of WL. Graph 1 shows total number of teeth with cracks on hand K-file and hand protaper file.

## DISCUSSION

Saunders et al^7^ suggested that dentinal cracks could lead to failure of the treatment because of microleakage and there is justified speculation as to whether these dentinal cracks would affect success over time.

The clinical significance of these cracks is speculative, but they may be of concern if there are residual bacteria that may colonize in these cracks. Over time, these cracks could also expand and contribute to leakage of the root-end filling and subsequent clinical failure.^8^ Instrumentation alone was found to significantly weaken the roots.^9,10^ Root canal instrumentation has the potential to induce dentinal damage and to generate cracks on the apical surface.^11^

Hand instruments perform complete shaping with significantly fewer rotations, but they take longer. Canal shape after preparation with hand files can be quite irregular.

From a fracture mechanics point of view, the presence of structural defects, cracks, or canal irregularities is likely to play a major role in determining fracture strength, because an applied stress may be exponentially amplified at the tip of those defects.

The basic sequence of protaper universal exhibits an advanced flute design that combines multiple tapers within the shaft, a convex triangular cross-sectional design, blades close to the noncutting pilot tip as well as an increasing chip space (space for the accumulation of debris) from tip to shaft. However, these effects could not be entirely eliminated, and differences among rotary NiTi instruments have been demonstrated (Liu R). With regard to the reported outcomes, it has to be stressed that different rake angles of instruments should reveal varying cutting efficacies.

In the present study, all samples were prepared to a size F2. This file has a tip size of 25 and a taper of 0.08 in the apical 3 mm of its length. The shaping files are designed for the purpose of enlarging the coronal and middle third of the root canal. The finishing files prepare the apical third after most of the dentin has been removed with the shaping files.

Root canal preparation is one of the most important steps in any root canal treatment.^1^ Root stresses generated from inside the root canal are higher in the apical region and along the canal wall than on the external surface. The pattern of stress distribution in the apical area could lead to the development of cracks and fracture propagation.^12^

The results of Adorno et al^13^ suggested that the cracks originating from the root canal might depend on the level of file insertion. When the WL reached the apical foramen, there was a higher risk of producing cracks. However, working 1 mm short of the apical foramen does not guarantee a “crack-free” result. They conclude that root canal preparation alone, regardless of the technique used, can potentially generate cracks on the apical root canal wall as well as the apical surface. Working 1 mm short of the apical foramen might produce fewer cracks in the apical region.

The clinician can, however, reduce crack susceptibility by maintaining the canal size as small as practical, and by striving for a smooth round canal without irregularities. In addition, clinicians can identify susceptible teeth before commencement of endodontic treatment based on root size and taper.^14^

## CONCLUSION

To date, many systems are used for cleaning and shaping of root canals, including hand and rotary systems, and all systems have their own advantages and disadvantages. So, this study was conducted to compare crack development during the use of different hand files, which showed cracks. It was found that maximum numbers of cracks were observed with hand protaper files compared with hand K-file at the WL and 1 mm short of WL.
